# Mothers with Serious Mental Illness: Their Experience of “Hitting Bottom”

**DOI:** 10.5402/2011/708318

**Published:** 2011-04-13

**Authors:** Phyllis Montgomery, Sharolyn Mossey, Patricia Bailey, Cheryl Forchuk

**Affiliations:** ^1^School of Nursing, Laurentian University, Ramsey Lake Road, Sudbury, ON, Canada P3E 2C6; ^2^Lawson Health Research Institute, Arthur Labatt Family School of Nursing, Faculty of Health Sciences, University of Western Ontario, 1151 Richmond Street, Health Sciences Addition, H4A, London, ON, Canada N6A 5C1

## Abstract

This study sought to understand the experience of “hitting bottom” from the perspective of 32 mothers with serious mental illness. Secondary narrative analysis of 173 stories about experiences related to hitting bottom were identified. Enactment of their perceived mothering roles and responsibilities was compromised when confronted by the worst of illness. Subsequent to women's descent to bottom was their need for a timely and safe exit from bottom. An intense experience in bottom further jeopardized their parenting and treatment self-determination and, for some, their potential for survival. The results suggest that prevention of bottom is feasible with early assessment of the diverse issues contributing to mothers' vulnerabilities. Interventions to lessen their pain may circumvent bottom experiences. Healing necessitates purposeful approaches to minimize the private and public trauma of bottom experiences, nurture growth towards a future, and establish resources to actualize such a life.

## 1. Introduction

Women diagnosed with serious mental illness (SMI) are just as likely to parent as other adult women in the general population. Research shows that this group of women have normal fertility rates [1] and higher rates of induced abortion [2]. Studies, however, report conflicting findings about the mean number of offspring for women with SMI. While some comparison studies report that women with SMI bear fewer than the average number of children [3], others show the opposite result [1]. This difference may partially be explained by women's age-at-first-diagnosis. That is, earlier age-at-first-diagnosis may contribute to a smaller mean number of offspring [4].

Globally, there is increasing awareness of the parenting status of mental health service users. It is estimated that approximately 60% of women with enduring mental health issues, internationally, have dependent children [5]. In both the United States and the United Kingdom, research shows that approximately one third of the female mental health services users are parents with dependent children [6–8]. In Canada, one in every ten children or 12.1% of all children under the age of 12 lives with a parent that had at least one psychiatric disorder in the previous 12 months [9]. Furthermore, only about one quarter of these parents reported receiving recent mental health services. 

The expanding research on parents with SMI remains largely focused on maternal role challenges in relation to the presence of illness. Some researchers suggest that families led by these women are among the most vulnerable and unsupported in communities [10–13]. As a group, they often contend with a range of health and social disadvantages that are compounded by illness. Such challenging circumstances include comorbid conditions [14, 15], poverty [16], discrimination [17], lack of social support [18], lack of health and parenting information [19], inadequate housing [20], trauma [21], and constant fear of their children's apprehension by Children's Protection Services [22]. 

According to Hollingsworth [23], a diagnosis of SMI is a “fast track” to the termination of parents' rights to the custody of their children. In a sample of 1954 mothers with SMI and other coexisting conditions, two-thirds were separated from all their dependent children [24]. Of the women who lived with at least one of their offspring, they reported significantly less stressful life events. Due to the stigma of mental illness and its associated socioeconomic implications, the juxtaposition for mothers with SMI relates to the reality that requests for help heighten the risk of child removal. It is therefore understandable that the lack of services or the feared implications of exposure by illness may contribute to mothers parenting without professional intervention. 

Without support, however, these mothers' parenting abilities may be jeopardized by the complex interplay of mothering, mothering contexts, illness, services needs, and resources. Bybee and associates [25] conducted a correlation study to explore factors affecting the day-to-day functioning of a group of 379 mothers with SMI. For mothers with low service involvement levels, functioning was inversely related to social stress. These researchers suggest that intensive services mediate the effects of stress, thereby allowing mothers to better attend to their parenting responsibilities. This strength-based orientation to parenting identifies that access to adequate resources fosters parenting success, regardless of a specific diagnostic label [16, 23]. According to Oyserman and associates [18].

Little research to date has sought out the voices of parents in disadvantaged circumstances… even though such research is critical if we are to understand the mechanisms underlying positive parenting (page 2505)

Although not all mothers with SMI experience negative parenting realities, for those that do, little is known about their subjective experiences. There are recent qualitative evidence reviews particular to parents with SMI [18, 26, 27]. The qualitative evidence presents emic perspectives from two groups: mothers and mental health service providers. From a maternal orientation, key findings include acknowledgement of role complexity, desire to minimize negative and optimize positive familial and individual outcomes, and identification of the need for significant trusted other [28–37]. A few studies present the perspective of those committed to servicing parents with SMI [38–41]. Overall, findings suggest the need for a range of individualized and purposeful material, emotional, educational, and relational supports to extend the benefits of existing treatment models.

In an effort to contribute to the developing understanding of women's experience, this paper explores the specific experience of “hitting bottom.” To date, the experience and language of hitting bottom in the nursing literature has been associated primarily with substance-related disorders [42] and to a lesser extent life-threatening chronic illnesses [43]. Therefore, for this particular group of women with a primary diagnosis of SMI, further examination of their “hitting bottom” experience is warranted. Further, from a women's agency and subjective orientation, knowledge about bottom experiences is imperative to the development of a strength-based, family-centered approach to well-being [16, 36].

## 2. Method

### 2.1. Design

The study design is a supplementary analysis, a type of secondary qualitative analysis [44, 45]. This approach is analogous to Thorne's [46] retrospective interpretation and analytic expansion types of secondary analysis. Supplementary analysis involves an in-depth examination of a theme or subset of data for the purpose of extending the primary work. Hitting bottom experiences were initially identified in a grounded theory study [31]. Bottom involved mothers' awareness of their distance from their children, compounded by an uncertainty of how to protect their children from witnessing intense illness. In two related studies about maternal health, mothers with SMI told unprompted, compelling stories that contained patterns revealing their human experience of “bottom”. Given that the data from these three studies, conducted between 2000 and 2008, were not analyzed to explore this phenomenon, secondary analysis offers a new layer of understanding of bottom as experienced by mothers. As Kohler Riessman [47] stated, the issue is not whether subsequent analysis of data is “truer,” rather, reinterpretation “illuminates a layered complexity…there is never a single authorised meaning” (page 321).

### 2.2. Setting and Sample

All of the ethically approved primary studies were conducted in northeastern Ontario. A variety of specialized mental health services were accessed since not one of them provided specific parenting programs for persons with SMI. Using theoretical and purposive sampling, a total of 37 inpatient and community women participated in the original studies. Study inclusion criteria were English-speaking, mentally competent, under a psychiatrist's care for a SMI as defined by Ministry of Health [48], and a mother of at least one child between the ages of two and sixteen years, either living with or separate from them.

### 2.3. Data Collection and Analysis

One to six audio-taped semistructured interviews were the main data-gathering strategies in all studies. In this study, narrative analysis of stories rather than the original studies' approach of thematic analysis was chosen. Narrative analysis preserves rather than fragments participants' stories [49]. Further, stories are frequently identified in qualitative data as a way in which participants construct meaning. Through analysis of stories, understanding of the emic perspective is sought. To complete this study's analysis, an eclectic narrative analysis approach was developed by the third author [50]. It combines functional analysis model of individual stories [51] with Agar and Hobbs [52] strategy of identifying story coherence across interviews. 

**Figure 1:**
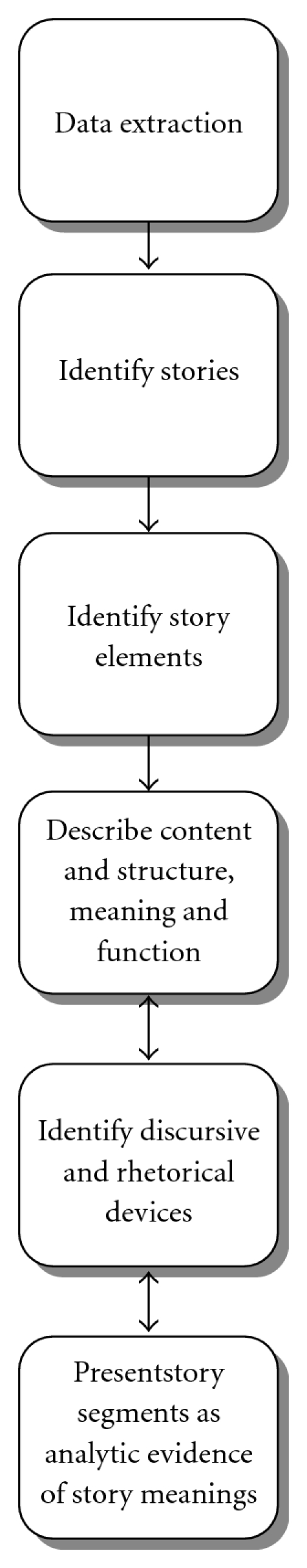
Narrative analysis process.

Figure 1 outlines the six iterative steps of the analysis process. Data about hitting bottom was extracted from each of the verbatim, unmarked transcripts and placed in a separate file. For this aggregate data set, the researchers individually deconstructed each story of bottom into its six elements inclusive of abstract, orientation, complicating action, resolution, evaluation, and coda [51]. Each element was compared across all stories to determine common, content, structure, function, and meaning. In addition, stories were examined for the inclusion of discursive and rhetorical devices [53] used to communicate complex and contentious points of view that might be outside typical experiences. Finally, the stories' meanings and functions were interpreted to develop an emic understanding of complex mothering realities in SMI.

## 3. Results

### 3.1. Storytellers

All of the women told stories of mothering and illness. Thirty-two of them specifically described a hitting bottom experience. The age range of these mothers was 19 to 38 years. Seventeen women lived with the biological fathers of the children. Twenty two mothers lived with their children, and of the 10 mothers separated from their children, all continued to keep contact with them. The women parented a total of 49 children ranging in age from two to 15 years. Each of the 32 mothers identified themselves as having a SMI diagnosis. During their length of illness, varying from three to twenty years, many reported receiving more than one mental health diagnoses. Several mothers acknowledged that they had been aware that “something wasn't quite right” for years prior to seeking psychiatric services. Other mothers said they became “suddenly” ill after giving birth. The most common primary psychiatric diagnosis they received during their course of illness was a major mood disorder. 

To represent the subjective understanding of bottom as expressed by the storytellers, the findings are presented in two sections, stories of bottom and a case example. First, the content and structure components of the 173 stories of hitting bottom are identified as the worst of illness, the escape imperative, and the consequences. Then, the meaning and function of the stories are outlined separately. Each of these components is illustrated by story excerpts. The inclusion of the symbol “/” denotes pauses often between ideas or phrases. Each excerpt is coded by an assigned participant identifier, for example, P1. To present a unified account of bottom, a case example inclusive of all story components concludes the findings. This format of presentation explicates the researchers' analytic logic, a reflection of fundamental credibility of this study [49, 54].

### 3.2. Stories of Bottom

#### 3.2.1. Content and Structure

Across the data base, the women told stories of hitting bottom contextualized by overwhelming illness that impeded enactment of their perceived roles and responsibilities as mothers. Three common content and structural components were identified in these stories. Mothers consistently talked of bottom as what they conceived to be the worst of illness. Second, mothers described their private internal dialogue as they searched for an exit from bottom with a limited range of possibilities. This structural and content component of the bottom stories was labelled as the escape imperative. Finally, these stories also included retrospective descriptions of the implications of being at bottom for themselves and their children. This final structural and content constituent was labelled as the consequences.

#### 3.2.2. The Worst of Illness

Women told stories characterizing the bottom experience as the worst possible illness reality. This story structure included three content areas: imposed kinetic force, unrecognizable self, and disaffiliation.

#### 3.2.3. Imposed Kinetic Force

Mothers attributed an actual or metaphorical motion to the worst of illness. As such, they explained an inescapable, often unexpected, and rapid descent to bottom as they found themselves “sliding” or “spiralling” out of control, “rolling down a hill”, and “falling, falling, falling”. This motion precipitated “losing touch”, “hitting hard” and finding themselves “at the end of their rope” in a “tunnel”. Excerpts of complete bottom stories which represent the kinetic experience of the worst of illness are as follows.

P2 I started sliding and sliding and I knew I won't be able to last much longer.
P23 I'd just be sitting here and just doing nothing and all of a sudden-boom. And it snowballed down from there.

#### 3.2.4. Unrecognizable Self

Women described the worst of illness experiences as intolerable affective distress compounded by an impaired ability to comprehend their circumstances. The emotional and cognitive alterations perceived by these women contributed to a difficulty in self-recognition within the worst of illness. This surreal reality was portrayed in phrases such as “it's not me,” “cannot live like this,” “cannot cope anymore,” and “life just does not seem real”.

P16 You do not think. You do not realize. You do not think you're broken in it.
P21 It's emotional torture/I know that/I equate it to being a homeless person living out of a cardboard box in the middle of a winter. It's minus 30. I have no shoes. No coat. This is how I felt at bottom-naked.
P24 Oh I was terrified. I did not know where I was or what was there. There was no help. Oh my God, my mind went insane. I got so confused.

#### 3.2.5. Disaffiliation

Women's accounts included the interplay of multiple familial, economic, health, and social service stressors. These external pressures combined with their SMI trajectory exacerbates real or perceived dissociation from others during the worst of illness. This disconnectedness from significant others and loss of typical role functioning served to “isolate” and “embarrass” them.

P19 Like they (Children's Services) just took the kids away/My mom did not want me to live with her because she had my (children) and we were fighting all the time.
P29 It is more about lost. I was just lost. Just feeling lost and not knowing where I belonged or who loved me.

#### 3.2.6. The Escape Imperative

Within the bottom stories, all women spoke about an intensive internal dialogue about an escape from bottom. This self-talk focused on their critical need to alleviate their suffering at bottom. Determined to search for an exit, they identified that there were “no easy answers” especially in the context of their diminishing physical, emotional, cognitive, and social resources. In such “devastating,” “discouraging,” “destructive,” and “cruel” circumstances many women considered the merit, pragmatics, and implications of suicide for themselves and significant others, most importantly their children as a way out of bottom.

P4 If want the best for my loved ones than maybe I need to disappear in the sense that yeah/ok/finish it off then. I may be doing more harm to my children and to my loved ones by being here than not being here at all. So basically that was the black and white of the situation at that point. I was incessantly tired, and started thinking very negatively and not being able to see beyond it you know. I want the best for my family.
P7 I could just die. I'm dying. I was just thinking of dying/if I could just die in my sleep. I would not do anything drastic/I thought of just dying/die of natural causes.
P10 I had no choice left. I thought lots about it. Suicide is not just about being unable to handle it anymore/it is more about not being able to live like this no more.

#### 3.2.7. The Consequences

The final structural component of the bottom stories was “the consequences” of living through this experience for their children and themselves as both a person and a mother. As mothers, they shared a primal sense of parental responsibility to protect their children regardless of the circumstances. Many women shared recollections of their struggle to fulfill this obligation in the midst of bottom. Although they expended effort to “pretend that things were OK,” they spoke of “tremendous guilt” associated with the “kids seeing suffering,” and “illness invading their (children's) lives”. Many women spoke of being ashamed that they experienced bottom and were displaced from their responsibilities as mother. Subsequent to the bottom experience, many women expressed a perceived sense of heightened familial and community surveillance and scrutiny of their mothering competency. For some women, loss of temporary or permanent custody was an imposed outcome of the bottom.

P1 I lost my joint custody.
P2 Ah (my child) feels abandoned/feels rejected/unloved and all it does to me is just/it adds more/I feel guilty as it is/it just adds to the guilt.
P5 You are second judging yourself and then you have others second judging you.
P6 I do not want the kids to see me suffer and worry about what effect it will have. I do not want them to see that or to know that. Why should, why should it invade their lives? (silence) I do not think it should. The kids they need just to be kids and then there's a need to grow up.
P27 (My child) is my main priority and God knows how long it is going to take me to get well in the hospital. There is no way I want (my child) in the hands of (child services). Because once it goes that far/there is more chance/more likely that you'll never get them back.

#### 3.2.8. Meaning

All of the women spoke of the importance and value of their role as mothers. In the context of bottom, the mother-child relationship became increasingly tenuous. The heightened intensity of the illness circumstances jeopardized their self-care practices and frequently compromised their ability to mother. Insight regarding the complexity of their descent to bottom, and their experiences while at bottom began to emerge only after escape from bottom. Mothers acknowledged that despite their best efforts to protectively conceal their illness circumstances, this was no longer possible. This illness-imposed impediment to their mothering role was associated with an intense private sense of guilt and contributed to worsening symptoms.

P11 You have so much pain you do not know where it goes so you turn it inward on yourself.

At bottom, these women knew that others questioned their fitness to mother. They feared losing their parental right to custody as a result of their illness-imposed bottom-experience. Their bottom stories illustrated their sense of powerless as both a person and a mother.

P2 (My child) could do a lot better. (My child) deserves a lot better.
P28 It's the people you know who try to get help that get their kids taken away from them.

A prolonged period or intense experience at bottom threatened women's self-determination in treatment, parenting involvement, and potentially survival. A timely and dignified escape from bottom was desired. For women survival through and from bottom was often influenced by perceived implications on their children.

P6 It's too hard/too hard. I feel sometimes there is no hope/You know I still feel that I will be around. That's a scary thought/very/very scary thought.
P17 If my (child) is not ok then I'm not.
P18 I love my children too much/they were the only reason I wanted to live.

Being caught in the “horror” of bottom, often void of resources, however, they were unable to actualize a “life without suffering” for themselves with their children. A reality sought but not achievable in the context of bottom.

#### 3.2.9. Function

The bottom stories demonstrate the impossibility of balancing responsibilities for self and others in the context of one's worst illness reality. Although bottom compromised the women's preferred mothering for a circumscribed period of time, it also sustained self and public doubt regarding their credible identity and capacity as mothers beyond bottom. These stories suggest that there is a need to create a safe context in which women may seek and receive early supportive care with a heightened likelihood of retaining their status as mother. Identifying and addressing the range of health and social circumstances that contributed to their vulnerabilities may lessen women's fragility and despair. Such issues, unattended at both a personal and social level, can accelerate movement to bottom, suspend women in bottom, and impede their safe exist from bottom.

### 3.3. Case Example

The case example outlined in Table 1 demonstrates inclusiveness of the above content, structure, meaning, and functional components of the bottom stories. P22 is a divorced mother living with her 12-year-old child. P22 had been diagnosed with schizophrenia 11 years prior to the initial data collection interview. The following narrative was told by P22 in response to the interviewer's request to elaborate on her expressed “not wanting or wishing to wake up in the morning” in relation to her discussion about bottom. Her expanded response acknowledged her bottom experience as distinct from suicidal ideation through her intent to not only escape her immediate circumstances, but re-establish agency.

## 4. Discussion

The bottom stories shared by the women in this study emphasized mothering in the context of overwhelming circumstances. When the “uncontrollable” force of illness disrupted their relationship with their children, their perceived experience of psychological pain was maximized. Within this context, hitting bottom was inevitable especially in the absence of comprehensive and appropriate mitigating resources. As such, there is an obligation for others, including health care professionals, to develop an awareness of each woman's unique indicators that may precipitate a descent to bottom. An informed understanding of such cues may provide an opportunity to develop an individualized woman-centered purposeful plan to prevent the intensity of the “fall” to bottom or ultimately, a complete avoidance of a bottom experience. Recent evidence [16, 29, 30, 41] reinforces that strengths-based, interagency support has the potential to make a positive difference for maternal mental health. By extension, this emphasizes the need for psychiatric mental health nurses to enhance their competency in delivering health-promoting family-focused interventions [11] as early as the prenatal period [55]. 

The experience for these women became increasingly complicated as their illness circumstances reciprocally exacerbated the challenges inherent in the mothering role to and at bottom. Health professionals who focus exclusively on acute symptoms without consideration of the mothering role may not appreciate the depth of the pain nor the potential strengths and motivations that will assist the woman to exit bottom safely. Hitting bottom may be a key point for intervention since these women described exhaustion and lack of trust regarding their internal resources or interpersonal connections to improve their situation. Unable to advocate for themselves at bottom may be liken to Broucek's [56] self-objectification in shame.

…is as if the ground under one's feet were giving way; depth and spatial relationships may have altered and one's place in space uncertain, resulting in a kind of vertigo. Such experiences dramatically illustrate to what extent the reality of our everyday phenomenal world is dependent on an intact sense of self and an intact set of interpersonal coordinates and not simply on an intact brain (page 40)

In the event that a bottom experience is not mitigated, there is an urgent need to minimize the trauma of a woman's experience. It is essential to provide guidance toward a safe exit that is not only desirable but achievable. A woman's contemporaneous planning for exit from bottom is difficult given that agency may be affected by the presence of acute symptoms. Early acknowledgement and ongoing validation of a woman's needs as mother is important to the creation of a genuine supported passage out of bottom. Innovative interventions to preserve agency and sense of meaningful connections to family involves affirming and strengthening women's competencies while cognizant of the oppressive presence of socially constructed unrealistic ideals of motherhood. [10, 19, 35] Such approaches may lessen the time and acuity of distress associated with bottom.

Once exit has been achieved, it is essential that women have an opportunity to talk through their experience in the context of a safe receptive relationship. The processing of the bottom experiences may allow for an increased understanding concerning the complexity of their descent to bottom and decrease their sense of guilt. Further, a purposeful debriefing may be conducive to healing thereby enhancing capacity for coping with the precipitating events and emerging consequences of bottom. The integration of a realistic insight about bottom is fundamentally linked to establishing a future characterized by well-being. Noh [57] asserted that responsibility in healing involves the acknowledgment of a continuum of women's competencies shaped by social, cultural, and familial supports. In the void of bottom, the women in this study emphasize the need to recognize their competency with appropriate resources.

A commonly identified limitation associated with secondary analyses relates to the assertion that qualitative data is co-constructed during the original study and thereby, limiting reuse for other purposes [45, 46]. The concerns about data reuse may be countered in this study given women's spontaneous telling of bottom stories in the original investigations. This emergent complex phenomenon warranted focused exploration by the primary researchers to more fully identify the salient features of bottom for mothers with SMI [45]. This secondary analysis generated a refined understanding of bottom that remains coherent with the preliminary conception in the original studies [31].

## 5. Conclusions

Thirty-two women with SMI, involved in three qualitative studies, told a total of 173 stories about experiences related to hitting bottom. These stories suggest that there is a need to create a safe context for women to receive early intervention to maximize their capacities and stability. Further, the results suggest that mental health providers must identify the diverse issues contributing to mothers' vulnerabilities in an effort to lessen their fragility and pain. Healing necessitates multiple approaches to address the private and public trauma of bottom experiences while nurturing their development. Preventative supportive family approaches are recommended given the burdensome legacy of bottom.

## Figures and Tables

**Table 1:** A Case Study.

Story element	Excerpt
Abstract	With me, hitting bottom was/(sigh) not necessarily wanted to commit suicide/but thinking about (child's name)/cause I was really thinking about (child's name). If I commit suicide what will happen to her? What will happen to me? I did not/I did not want to go past the point of no return. I wanted to make sure I knew what I was doing and whether I was willing to face the consequences whatever that means.

Orientation	Being a mother, I know that uhmm/I mean/I wouldn't be the person I am today/uhmm/the good parts of me I mean if I did not have (my child). I feel that being her mom, uhmm makes me a better person in a lot of ways although I have lots of way to go.

Complicating action	Ever since (child's name) been little/and I've been uhmm struggling with illness uhmm it's definitely gradual uhmm it starts out with me missing one (with emphasis) dosage of medication here or these/so uhmm if I miss a dosage here or there I start to be a little more confrontational/a little bit more defensive/a little more paranoid/what are they saying about me. It is a spiral, it's kind of like a snowball rolling down a hill/it starts small small small/It gets bigger bigger bigger and then finally it's a huge (with emphasis). Nothing can stop the illness now. It is made up of worries responsibilities uhmm—uhmm/emotional torture.
	I made it known that if I got real sick that (child's name) would go to stay with my friend and her husband. I was thinking that would be best for her because of the fact of her having to live with a mom who was so sick.
	I've always begged my friend/I've always said “you know (friend's name) really I'm a bad mom (with emphasis). I cannot handle this. (My child) is a great kid. (My child) needs better than me”.

Resolution	My friend always said to me “You are fine. You are fine. You can get through this.” She does not take (child's name).

Evaluation	I realized/and think that (my friend) knows what she is doing when she does not take (my child)/like I said being with my child makes me able to be a better person holding back from doing something stupid.

Coda	I just came out of that hole that I was in. I understand it much better now. I can actually talk myself through a little bit about my past lives. I'm not all illness you know. There are parts of me that are not illness.
